# Broken Limbs

**Published:** 1870-10

**Authors:** 


### BROKEN LIMBS

Have resulted from thoughtless persons throwing the peelings of fruit or other article on the sidewalk ; a peach-stone may occasion the same thing, or the rind of a lemon or tomato ; it is uncomely even to expectorate on the sidewalk ; step to the curb-stone ; avoidance is not the whole duty of a good man, if you see anything on the sidewalk which might cause a fall to a child or tottering age or invalid. In going down Fulton street, we observed two women with market baskets, one following the other ; each of them stepped aside, and with a quick motion of the foot, brushed banana skins from the pavement. There was as much pure benevolence in that little act as in the bestowal of thousands from the strong box of the millionaire.

